# Characterisation of age and polarity at onset in bipolar disorder

**DOI:** 10.1192/bjp.2021.102

**Published:** 2021-12

**Authors:** Janos L. Kalman, Loes M. Olde Loohuis, Annabel Vreeker, Andrew McQuillin, Eli A. Stahl, Douglas Ruderfer, Maria Grigoroiu-Serbanescu, Georgia Panagiotaropoulou, Stephan Ripke, Tim B. Bigdeli, Frederike Stein, Tina Meller, Susanne Meinert, Helena Pelin, Fabian Streit, Sergi Papiol, Mark J. Adams, Rolf Adolfsson, Kristina Adorjan, Ingrid Agartz, Sofie R. Aminoff, Heike Anderson-Schmidt, Ole A. Andreassen, Raffaella Ardau, Jean-Michel Aubry, Ceylan Balaban, Nicholas Bass, Bernhard T. Baune, Frank Bellivier, Antoni Benabarre, Susanne Bengesser, Wade H Berrettini, Marco P. Boks, Evelyn J. Bromet, Katharina Brosch, Monika Budde, William Byerley, Pablo Cervantes, Catina Chillotti, Sven Cichon, Scott R. Clark, Ashley L. Comes, Aiden Corvin, William Coryell, Nick Craddock, David W. Craig, Paul E. Croarkin, Cristiana Cruceanu, Piotr M. Czerski, Nina Dalkner, Udo Dannlowski, Franziska Degenhardt, Maria Del Zompo, J. Raymond DePaulo, Srdjan Djurovic, Howard J. Edenberg, Mariam Al Eissa, Torbjørn Elvsåshagen, Bruno Etain, Ayman H. Fanous, Frederike Fellendorf, Alessia Fiorentino, Andreas J. Forstner, Mark A. Frye, Janice M. Fullerton, Katrin Gade, Julie Garnham, Elliot Gershon, Michael Gill, Fernando S. Goes, Katherine Gordon-Smith, Paul Grof, Jose Guzman-Parra, Tim Hahn, Roland Hasler, Maria Heilbronner, Urs Heilbronner, Stephane Jamain, Esther Jimenez, Ian Jones, Lisa Jones, Lina Jonsson, Rene S. Kahn, John R. Kelsoe, James L. Kennedy, Tilo Kircher, George Kirov, Sarah Kittel-Schneider, Farah Klöhn-Saghatolislam, James A. Knowles, Thorsten M. Kranz, Trine Vik Lagerberg, Mikael Landen, William B. Lawson, Marion Leboyer, Qingqin S. Li, Mario Maj, Dolores Malaspina, Mirko Manchia, Fermin Mayoral, Susan L. McElroy, Melvin G. McInnis, Andrew M. McIntosh, Helena Medeiros, Ingrid Melle, Vihra Milanova, Philip B. Mitchell, Palmiero Monteleone, Alessio Maria Monteleone, Markus M. Nöthen, Tomas Novak, John I. Nurnberger, Niamh O'Brien, Kevin S. O'Connell, Claire O'Donovan, Michael C. O'Donovan, Nils Opel, Abigail Ortiz, Michael J. Owen, Erik Pålsson, Carlos Pato, Michele T. Pato, Joanna Pawlak, Julia-Katharina Pfarr, Claudia Pisanu, James B. Potash, Mark H Rapaport, Daniela Reich-Erkelenz, Andreas Reif, Eva Reininghaus, Jonathan Repple, Hélène Richard-Lepouriel, Marcella Rietschel, Kai Ringwald, Gloria Roberts, Guy Rouleau, Sabrina Schaupp, William A Scheftner, Simon Schmitt, Peter R. Schofield, K. Oliver Schubert, Eva C. Schulte, Barbara Schweizer, Fanny Senner, Giovanni Severino, Sally Sharp, Claire Slaney, Olav B. Smeland, Janet L. Sobell, Alessio Squassina, Pavla Stopkova, John Strauss, Alfonso Tortorella, Gustavo Turecki, Joanna Twarowska-Hauser, Marin Veldic, Eduard Vieta, John B. Vincent, Wei Xu, Clement C. Zai, Peter P. Zandi, Arianna Di Florio, Jordan W. Smoller, Joanna M. Biernacka, Francis J. McMahon, Martin Alda, Bertram Müller-Myhsok, Nikolaos Koutsouleris, Peter Falkai, Nelson B. Freimer, Till F.M. Andlauer, Thomas G. Schulze, Roel A. Ophoff

**Affiliations:** Institute of Psychiatric Phenomics and Genomics (IPPG), University Hospital, LMU Munich, Germany; Department of Psychiatry and Psychotherapy, University Hospital Munich, Germany; and International Max Planck Research School for Translational Psychiatry, Germany; Center for Neurobehavioral Genetics, Semel Institute for Neuroscience and Human Behavior, University of California Los Angeles, USA; Department of Child and Adolescent Psychiatry/Psychology, Erasmus University Medical Centre–Sophia Children’s Hospital, the Netherlands; Division of Psychiatry, University College London, UK; Division of Psychiatric Genomics, Mount Sinai School of Medicine, USA; Division of Genetic Medicine, Department of Medicine, Vanderbilt Genetics Institute, Vanderbilt University Medical Center, USA; Department of Biomedical Informatics, Vanderbilt University Medical Center, USA; and Department of Psychiatry and Behavioral Sciences, Vanderbilt University Medical Center, USA; Alexandru Obregia Clinical Psychiatric Hospital, Bucharest, Romania; Department of Psychiatry and Psychotherapy, Charite – Universitätsmedizin, Germany; Analytic and Translational Genetics Unit, Massachusetts General Hospital, USA; and Stanley Center for Psychiatric Research, Broad Institute of MIT and Harvard, USA; Department of Psychiatry and Behavioral Sciences, SUNY Downstate Health Sciences University, USA; and VA NY Harbor Healthcare System, USA; Department of Psychiatry and Psychotherapy, Philipps-University Marburg, Germany; Department of Psychiatry and Psychotherapy, Philipps-University Marburg, Germany; and Center for Mind, Brain and Behavior (CMBB), Germany; Institute for Translational Psychiatry, Westfälische Wilhelms-Universität Münster, Germany; and Institute for Translational Neuroscience, University of Münster, Germany; International Max Planck Research School for Translational Psychiatry, Germany; and Max Planck Institute of Psychiatry, Germany; Department of Genetic Epidemiology in Psychiatry, Central Institute of Mental Health, Medical Faculty Mannheim, Heidelberg University, Germany; Institute of Psychiatric Phenomics and Genomics (IPPG), University Hospital, LMU Munich, Germany; Department of Psychiatry and Psychotherapy, University Hospital Munich, Germany; and Centro de Investigación Biomedica en Red de Salud Mental (CIBERSAM), Spain; Division of Psychiatry, University of Edinburgh, UK; Department of Clinical Sciences, Medical Faculty, Umeå University, Sweden; Institute of Psychiatric Phenomics and Genomics (IPPG), University Hospital, LMU Munich, Germany; and Department of Psychiatry and Psychotherapy, University Hospital Munich, Germany; Department of Clinical Neuroscience, Centre for Psychiatry Research, Karolinska Institutet, Sweden; Department of Psychiatric Research, Diakonhjemmet Hospital, Norway; and NORMENT Centre, Division of Mental Health and Addiction, Institute of Clinical Medicine, University of Oslo, Norway; Division of Mental Health and Addiction, Oslo University Hospital, Norway; and NORMENT Centre, Inst of Clinical Medicine, University of Oslo, Norway; Department of Psychiatry and Psychotherapy, University Medical Center Göttingen, Germany; NORMENT Centre, Inst of Clinical Medicine, University of Oslo, Norway; and Division of Mental Health and Addiction, Oslo University Hosptial, Norway; Unit of Clinical Pharmacology, University Hospital Agency of Cagliari, Italy; Faculty of medicine, University of Geneva, Switzerland; and Department of Psychiatry, Psychosomatic Medicine and Psychotherapy, University Hospital Frankfurt, Germany; Department of Psychiatry, Psychosomatic Medicine and Psychotherapy, University Hospital Frankfurt, Germany; Department of Psychiatry, University of Münster, Germany; Department of Psychiatry, Melbourne Medical School, The University of Melbourne, Australia; The Florey Institute of Neuroscience and Mental Health, The University of Melbourne, Australia; and Discipline of Psychiatry, Adelaide Medical School, The University of Adelaide, Australia; Universite de Paris, France; INSERM UMRS 1144, France; and DMU Neurosciences, GHU Lariboisière Fernand Widal, Departement de Psychiatrie, APHP, France; Hospital Clinic, University of Barcelona, IDIBAPS, CIBERSAM, Spain; Department of Psychiatry and Psychotherapeutic Medicine, Medical University Graz, Austria; Psychiatry, University of Pennsylvania, USA; Psychiatry, UMC Utrecht Brain Center, the Netherlands; Department of Psychiatry, Stony Brook University, USA; Institute of Psychiatric Phenomics and Genomics (IPPG), University Hospital, LMU Munich, Germany; Psychiatry, University of California San Francisco, USA; Department of Psychiatry, McGill University, Canada; Department of Biomedicine, University of Basel, Switzerland; Institute of Human Genetics, University of Bonn, School of Medicine & University Hospital Bonn, Germany; Institute of Medical Genetics and Pathology, University Hospital Basel, Switzerland; and Institute of Neuroscience and Medicine (INM-1), Research Centre Julich, Germany; Discipline of Psychiatry, University of Adelaide, Australia; and Bazil Hetzel Institute, Australia; Department of Psychiatry & Trinity Translational Medicine Institute, Trinity College Dublin, Ireland; University of Iowa Hospitals and Clinics, USA; Medical Research Council Centre for Neuropsychiatric Genetics and Genomics, Division of Psychological Medicine and Clinical Neurosciences, Cardiff University, UK; Translational Genomics, USC, USA; Department of Psychiatry and Psychology, Mayo Clinic, USA; Department of Translational Research, Max Planck Institute of Psychiatry, Germany; Department of Psychiatric Genetics, Poznan University of Medical Sciences, Poland; Institute for Translational Psychiatry, Westfälische Wilhelms-Universität Münster, Germany; Institute of Human Genetics, University of Bonn, School of Medicine & University Hospital Bonn, Germany; and Department of Child and Adolescent Psychiatry, Psychosomatics and Psychotherapy, University Hospital Essen, University of Duisburg-Essen, Germany; Department of Psychiatry and Behavioral Sciences, Johns Hopkins University, USA; Department of Medical Genetics, Oslo University Hospital Ullevål, Norway; and NORMENT, Department of Clinical Science, University of Bergen, Norway; Department of Biochemistry and Molecular Biology, Indiana University School of Medicine, USA; NORMENT, Division of Mental Health and Addiction, Oslo University Hospital, Norway; Institute of Human Genetics, University of Bonn, School of Medicine & University Hospital Bonn, Germany; and Centre for Human Genetics, University of Marburg, Germany; Neuroscience Research Australia, Australia; and School of Medical Sciences, University of New South Wales, Australia; Nova Scotia Health Authority, Canada; Department of Psychiatry and Behavioral Neuroscience, University of Chicago, USA; and Department of Human Genetics, University of Chicago, USA; Psychological Medicine, University of Worcester, UK; Mood Disorders Centre of Ottawa, Canada; and Department of Psychiatry, University of Toronto, Canada; Mental Health Department, University Regional Hospital, Biomedicine Institute (IBIMA), Spain; Cell Biology, SUNY Downstate Medical Center College of Medicine, USA; and Institute for Genomic Health, SUNY Downstate Medical Center College of Medicine, USA; Universite Paris Est Creteil, France; and INSERM U 955, Neuropsychiatrie Translationnelle, France; Department of Psychiatry and Neurochemistry, Institute of Neuroscience and Physiology, The Sahlgrenska Academy at the University of Gothenburg, Sweden; Department of Psychiatry, Icahn School of Medicine at Mount Sinai, USA; Department of Psychiatry, University of California San Diego, USA; Department of Psychiatry, University of Toronto, Canada; The Campbell Family Mental Health Research Institute, Centre for Addiction and Mental Health, Canada; and Institute of Medical Science, University of Toronto, Canada; Department of Psychiatry, Psychosomatic Medicine and Psychotherapy, University Hospital Frankfurt, Germany; and Department of Psychiatry, Psychotherapy and Psychosomatics, University Hospital Wurzburg, Germany; NORMENT Centre, Division of Mental Health and Addiction, Oslo University Hosptial, Norway; Department of Psychiatry and Neurochemistry, Institute of Neuroscience and Physiology, The Sahlgrenska Academy at the University of Gothenburg, Sweden; and Department of Medical Epidemiology and Biostatistics, Karolinska Institutet, Sweden; Department of Psychiatry and Behavioral Sciences, Howard University Hospital, USA; Neuroscience, Janssen Research & Development, USA; Department of Psychiatry, University of Campania ‘Luigi Vanvitelli’, Italy; Department of Psychiatry, Icahn School of Medicine at Mount Sinai, USA; and Department of Genetics & Genomics, Icahn School of Medicine at Mount Sinai, USA; Unit of Psychiatry, Department of Medical Sciences and Public Health, University of Cagliari, Italy and Department of Pharmacology, Dalhousie University, Canada; Research Institute, Lindner Center of HOPE, USA; Department of Psychiatry, University of Michigan, USA; Institute for Genomic Health, SUNY Downstate Medical Center College of Medicine, USA; NORMENT Centre, Division of Mental Health and Addiction, Institute of Clinical Medicine and Diakonhjemmet Hospital, University of Oslo, Norway; and Division of Mental Health and Addiction, Oslo University Hospital, Norway; Psychiatric Clinic, Alexander University Hospital, Bulgaria; School of Psychiatry, University of New South Wales, Australia; Department of Medicine, Surgery and Dentistry ‘Scuola Medica Salernitana’, University of Salerno, Italy; Institute of Human Genetics, University of Bonn, School of Medicine & University Hospital Bonn, Germany; National Institute of Mental Health, Czech Republic; Psychiatry, Indiana University School of Medicine, USA; Department of Psychiatry, Dalhousie University, Canada; Department of Psychiatry, University of Toronto, Toronto, Canada; and Centre for Addiction and Mental Health, Toronto, Canada; Department of Biomedical Science, Section of Neuroscience & Clinical Pharmacology, University of Cagliari, Italy; Department of Psychiatry and Behavioral Sciences, Emory University, USA; Department of Psychiatry, Geneva University Hospitals, Switzerland; Montreal Neurological Institute, Canada and Department of Neurology, McGill University, Canada; Department of Psychiatry, Rush Medical College, USA; Discipline of Psychiatry, University of Adelaide, Australia; and Northern Adelaide Mental Health Service, SA Health, Australia; Psychiatry and the Behavioral Sciences, University of Southern California, USA; Department of Psychiatry, Dalhousie University, Canada; and Department of Biomedical Science, Section of Neuroscience & Clinical Pharmacology, University of Cagliari, Italy; Department of Psychiatry, University of Perugia, Italy; Department of Psychiatry, McGill University, Canada; and Douglas Institute, McGill University, Canada; Dalla Lana School of Public Health, Biostatistics Division, University of Toronto, Canada; Department of Psychiatry, University of Toronto, Canada; The Campbell Family Mental Health Research Institute, Centre for Addiction and Mental Health, Canada; Institute of Medical Science, University of Toronto, Canada; Laboratory Medicine and Pathobiology, University of Toronto, Canada; and Harvard T.H. Chan School of Public Health, USA; Psychiatric and Neurodevelopmental Genetics Unit, Department of Psychiatry and Center for Genomic Medicine, Massachusetts General Hospital, USA; and Stanley Center for Psychiatric Research, Broad Institute of MIT and Harvard, USA; Department of Psychiatry and Psychology, Mayo Clinic, USA; and Department of Health Sciences Research, Mayo Clinic, USA; Human Genetics Branch, Intramural Research Program, National Institute of Mental Health, USA; National Institute of Mental Health, Czech Republic; and Department of Psychiatry, Dalhousie University, Canada; Max Planck Institute of Psychiatry, Germany; Department of Psychiatry and Psychotherapy, University Hospital Munich, Germany; Max Planck Institute of Psychiatry, Germany; and Institute of Psychiatry, Psychology and Neuroscience, Kings College London, UK; Department of Psychiatry and Psychotherapy, University Hospital Munich, Germany; Center for Neurobehavioral Genetics, Semel Institute for Neuroscience and Human Behavior, University of California Los Angeles, USA; and Human Genetics, University of California Los Angeles, USA; Department of Neurology, Klinikum rechts der Isar, School of Medicine, Technical University of Munich, Germany; Institute of Psychiatric Phenomics and Genomics (IPPG), University Hospital, LMU Munich, Germany; Department of Genetic Epidemiology in Psychiatry, Central Institute of Mental Health, Medical Faculty Mannheim, Heidelberg University, Germany; Department of Psychiatry and Psychotherapy, University Medical Center Göttingen, Germany; Department of Psychiatry and Behavioral Sciences, Johns Hopkins University School of Medicine, USA; and Department of Psychiatry and Behavioral Sciences, SUNY Upstate Medical University, USA; Center for Neurobehavioral Genetics, Semel Institute for Neuroscience and Human Behavior, University of California Los Angeles, USA; Human Genetics, University of California Los Angeles, USA; and Psychiatry, Erasmus University Medical Center, the Netherlands; Department of Biomedical Science, Section of Neuroscience & Clinical Pharmacology, University of Cagliari, Italy; and Unit of Clinical Pharmacology, University Hospital Agency of Cagliari, Italy

**Keywords:** Bipolar disorder, age at onset, polarity at onset, GWAS, polygenic score

## Abstract

**Background:**

Studying phenotypic and genetic characteristics of age at onset (AAO) and polarity at onset (PAO) in bipolar disorder can provide new insights into disease pathology and facilitate the development of screening tools.

**Aims:**

To examine the genetic architecture of AAO and PAO and their association with bipolar disorder disease characteristics.

**Method:**

Genome-wide association studies (GWASs) and polygenic score (PGS) analyses of AAO (*n* = 12 977) and PAO (*n* = 6773) were conducted in patients with bipolar disorder from 34 cohorts and a replication sample (*n* = 2237). The association of onset with disease characteristics was investigated in two of these cohorts.

**Results:**

Earlier AAO was associated with a higher probability of psychotic symptoms, suicidality, lower educational attainment, not living together and fewer episodes. Depressive onset correlated with suicidality and manic onset correlated with delusions and manic episodes. Systematic differences in AAO between cohorts and continents of origin were observed. This was also reflected in single-nucleotide variant-based heritability estimates, with higher heritabilities for stricter onset definitions. Increased PGS for autism spectrum disorder (β = −0.34 years, s.e. = 0.08), major depression (β = −0.34 years, s.e. = 0.08), schizophrenia (β = −0.39 years, s.e. = 0.08), and educational attainment (β = −0.31 years, s.e. = 0.08) were associated with an earlier AAO. The AAO GWAS identified one significant locus, but this finding did not replicate. Neither GWAS nor PGS analyses yielded significant associations with PAO.

**Conclusions:**

AAO and PAO are associated with indicators of bipolar disorder severity. Individuals with an earlier onset show an increased polygenic liability for a broad spectrum of psychiatric traits. Systematic differences in AAO across cohorts, continents and phenotype definitions introduce significant heterogeneity, affecting analyses.

## Background

Bipolar disorder is highly heritable and affects approximately 1% of the population. It has a recurrent or chronic course and is associated with psychosocial impairment and reduced functioning, and it is a leading cause of global disease burden.^1^ Individuals usually experience their first (hypo)manic or depressive episode of bipolar disorder in adolescence or early adulthood, but often they are not diagnosed until 5 to 10 years later,^2^ especially in individuals with an earlier age at onset (AAO) or a depressive index episode.^3^ Early illness onset is associated with a more severe disease course and greater impairment across a wide range of mental and physical disorders and is a useful prognostic marker.^4–7^ However, pathophysiological processes leading to a disorder are thought to begin long before the first symptoms appear.^8,9^ Investigating the factors contributing to age and polarity (i.e. either a (hypo)manic or depressive episode) at onset could thus improve our understanding of disease pathophysiology and facilitate development of personalised screening and preventive measures. Accordingly, AAO and polarity at onset (PAO) of bipolar disorder are considered as suitable phenotypes for genetic analyses.

Genome-wide association studies (GWASs) have improved our understanding of the genetic architecture of susceptibility to bipolar disorder; however, the genetic determinants of AAO and PAO remain largely unknown. Evidence suggests that patients with an early AAO carry a stronger genetic loading for bipolar disorder risk.^10^ For example, an earlier parental AAO increases familial risk for bipolar disorder and is one of the strongest predictors of 5-year illness onset in affected offspring.^10–12^ Previous research has described that a higher genetic risk burden for schizophrenia may be associated with earlier AAO of bipolar disorder,^13^ but this finding did not replicate.^14–16^ Moreover, a recent study did not find an association of bipolar disorder polygenic score (PGS) with AAO.^17^ Thus far, GWASs for age at bipolar disorder onset have been underpowered,^18,19^ and a study of 8610 patients found no significant evidence for a heritable component contributing to onset age.^13^ The PAO was shown to cluster in families,^20^ but the genetic architecture of PAO has not yet been investigated.

## Aims

To fill these knowledge gaps, we performed comprehensive analyses of AAO and PAO of bipolar disorder in the largest sample studied to date by (a) examining phenotype definitions and associations, (b) investigating whether the genetic load for neuropsychiatric disorders and traits contributes to AAO and PAO of bipolar disorder, and (c) conducting systematic GWASs.

## Method

References to published methods are listed in Supplementary Note 1 available at https://doi.org/10.1192/bjp.2021.102.

### Study samples

Participants with a bipolar disorder diagnosis, available genetic data and AAO information were selected from independent data-sets, including those previously submitted to the Psychiatric Genomics Consortium (PGC) Bipolar Disorder Working Group^13^ and the International Consortium on Lithium Genetics (ConLiGen).^21^ These consortia aggregate genetic data from many cohorts worldwide. Our analyses comprised 34 cohorts with 12 977 patients with bipolar disorder who have European ancestry from Europe, North America and Australia. For a description of sample ascertainment, see the Supplementary Material.

**Fig. 1:**
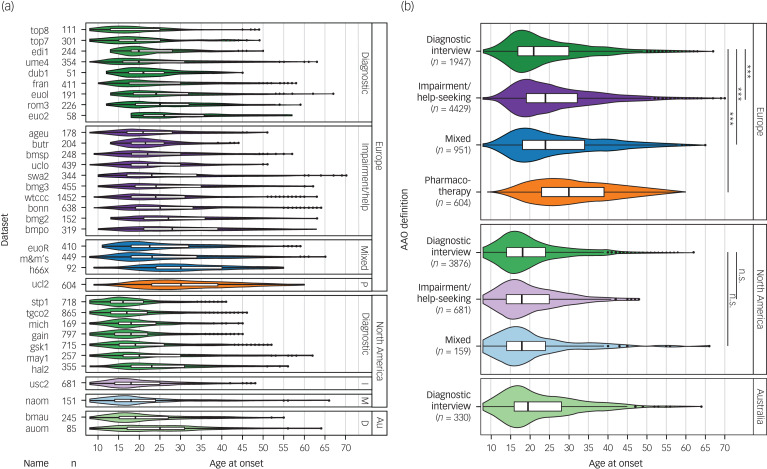
Differences between phenotype definitions and continents across the 34 data-sets used for discovery-stage genetic analyses. (a) The various data-sets used four different definitions for age at onset: diagnostic interview, impairment/help-seeking, pharmacotherapy and mixed. (b) The untransformed age at onset differed significantly between cohorts, depending on the phenotype definition used and the continent of origin. Au, Australia; Diagnostic, diagnostic interview; D, diagnostic interview; I, impairment/help-seeking; M, mixed; P, pharmacotherapy. n.s., not significant; *P* > 0.05; *** *P* < 0.001.

The authors assert that all procedures contributing to this work comply with the ethical standards of the relevant national and institutional committees on human experimentation and with the Helsinki Declaration of 1975, as revised in 2008. All procedures involving human patients were approved by the local ethics committees, and written informed consent was obtained from all patients. For details on the data-sets, including phenotype definitions and distributions, see Table 1, Fig. 1, and Supplementary Table S1.

**Table 1:** Sample characteristics of data-sets used in genetic analyses

GWAS stage, dataset	*n*	Continent	Diagnosis, % bipolar disorder type I	Gender, % male	AAO, median (MAD,^a^ range)	Definition of AAO	PAO,^b^ *n* (%)
Discovery							
wtccc	1452	Europe	89.53	36.85	24 (8.9, 9–63)	Impairment/help-seeking	
tgco2	865	North America	100	33.64	17 (5.93, 8–46)	Diagnostic interview	PAO-M: 316 (38.92); PAO-D: 496 (61.08)
gain	797	North America	100	48.06	18 (5.93, 8–45)	Diagnostic interview	PAO-M: 135 (18.57); PAO-D: 440 (60.52)
stp1	718	North America	100	44.01	16 (5.93, 8–41)	Diagnostic interview	PAO-M: 137 (19.08); PAO-D: 420 (58.5)
gsk1	715	North America	89.51	36.36	19 (7.51, 8–52)	Diagnostic interview	PAO-M: 102 (14.61); PAO-D: 395 (56.59)
usc2	681	North America	96.18	47.58	18 (7.41, 8–48)	Impairment/help-seeking	
bonn	638	Europe	99.84	47.34	25 (8.9, 9–64)	Impairment/help-seeking	
ucl2	604	Europe	100	44.37	30 (11.86, 9–60)	Pharmacotherapy	PAO-M: 47 (9.96); PAO-D: 209 (44.28)
bmg3	455	Europe	57.14	40.66	24 (10.38, 10–62)	Impairment/help-seeking	PAO-M: 43 (16.35); PAO-D: 159 (60.46)
m&m's	449	Europe	74.83	52.12	23 (10.38, 8–65)	Mixed	PAO-M: 73 (17.14); PAO-D: 238 (55.87)
uclo	439	Europe	100	39.86	22 (7.41, 8–51)	Impairment/help-seeking	PAO-M: 54 (14.25); PAO-D: 197 (51.98)
fran	411	Europe	77.62	41.36	22 (7.41, 10–58)	Diagnostic interview	
euoR	410	Europe	75.85	44.15	22 (9.64, 11–59)	Mixed	
hal2	355	North America	71.55	42.54	23 (8.9, 8–56)	Diagnostic interview	PAO-M: 102 (29.65); PAO-D: 213 (61.92)
ume4	354	Europe	69.21	37.85	20 (8.9, 8–63)	Diagnostic interview	PAO-M: 54 (14.25); PAO-D: 197 (51.98)
swa2	344	Europe	81.10	41.86	23 (10.38, 10–70)	Impairment/help-seeking	
bmpo	319	Europe	78.06	39.18	28 (11.86, 10–63)	Impairment/help-seeking	PAO-M: 41 (16.33); PAO-D: 150 (59.76)
top7	301	Europe	62.79	41.53	19 (7.41, 8–49)	Diagnostic interview	
may1	257	North America	100	45.14	20 (8.9, 8–62)	Diagnostic interview	PAO-M: 34 (13.23); PAO-D: 142 (55.25)
bmsp	248	Europe	94.76	45.56	22 (7.41, 9–57)	Impairment/help-seeking	PAO-M: 24 (10.04); PAO-D: 93 (38.91)
bmau	245	Australia	79.18	40.82	19 (7.41, 8–55)	Diagnostic interview	PAO-M: 46 (20.18); PAO-D: 125 (54.82)
edi1	244	Europe	99.18	42.62	20 (5.93, 13–50)	Diagnostic interview	
rom3	226	Europe	100	41.15	25 (10.38, 12–59)	Diagnostic interview	PAO-M: 91 (40.27); PAO-D: 134 (59.29)
butr	204	Europe	100	40.2	22 (5.19, 13–44)	Impairment/help-seeking	
euoI	191	Europe	74.87	31.41	24 (8.9, 13–67)	Diagnostic interview	PAO-M: 48 (27.43); PAO-D: 98 (56)
ageu	178	Europe	90.45	39.33	21 (7.41, 8–51)	Impairment/help-seeking	
mich	169	North America	100	31.36	18 (5.93, 8–45)	Diagnostic interview	PAO-M: 42 (24.85); PAO-D: 84 (49.7)
naom	159	North America	84.91	44.65	18 (7.41, 8–66)	Mixed	PAO-M: 30 (28.85); PAO-D: 51 (49.04)
bmg2	152	Europe	59.87	35.53	27 (10.38, 13–63)	Impairment/help-seeking	
top8	111	Europe	55.86	37.84	18 (7.41, 8–49)	Diagnostic interview	
h66x	92	Europe	82.61	36.96	30 (10.38, 9–55)	Mixed	
auom	85	Australia	88.24	45.88	25 (10.38, 8–64)	Diagnostic interview	
euo2	58	Europe	65.52	56.9	26 (8.9, 18–57)	Diagnostic interview	
dub1	51	Europe	100	54.9	21 (5.93, 12–45)	Diagnostic interview	
Summary	12 977		88.27	41.57	21 (8.9, 8–70)		PAO–M: 1435 (21.19); PAO-D: 3885 (57.36)
Replication							
ukwa1	1156	Europe	75.17	38.15	23 (8.9, 8–74)	Impairment/help-seeking	
dutch	468	Europe	100	42.31	28 (10.38, 11–63)	Pharmacotherapy	
jst5	186	North America	100	53.23	16 (7.41, 8–51)	Unknown	
colo	176	South America	90.34	31.82	20 (11.86, 8–52)	Diagnostic interview	
bmrom	126	Europe	100	42.86	24 (8.9, 12–56)	Diagnostic criteria	
bdtrs	125	Europe	64	45.6	28 (13.34, 8–65)	Impairment/help-seeking	
Summary	2237		84.40	40.46	24 (10.38, 8–74)		
All data	15 214		86.26	41.41	22 (8.9, 8–74)		

GWAS, genome-wide association study; AAO, age at onset; MAD, median absolute deviation, PAO, polarity at onset; PAO-M, mania/hypomania before depression; PAO-D, depression before mania/hypomania.

a. We calculated the median absolute deviation using 1.4826 as constant.

b. We defined three categories of polarity at onset: PAO-M, mania/hypomania before depression; PAO-D, depression before mania/hypomania; and PAO-X, mixed. PAO was not available for all patients. The table presents the PAO-M and PAO-D subgroups and their percentage within the individual cohorts.

### Definition of AAO

The definition of age at bipolar disorder onset differed by cohort. To enhance cross-cohort comparability, we grouped the definitions into four broad categories as follows (Supplementary Table S1).

- Diagnostic interview: age at which the patient first experienced a (hypo)manic, mixed or major depressive episode according to a standardised diagnostic interview.
- Impairment/help-seeking: age at which symptoms began to cause subjective distress or impaired functioning or at which the patient first sought psychiatric treatment.
- Pharmacotherapy: age at first administration of medication.
- Mixed: a combination of the above-mentioned definitions.

Across definitions, participants younger than 8 years at onset were excluded (n = 279) because of the uncertainty about the reliability of retrospective recall of early childhood onset. The distribution of AAO was highly skewed and differed considerably between the cohorts (Table 1 and Fig. 1). Therefore, we transformed AAO in each cohort by rank-based inverse-normal transformation and used this normalised variable as the primary dependent variable in all genetic analyses. To facilitate interpretability of effect sizes, we also report results of the corresponding untransformed AAO.

### Definition of PAO

For each cohort, PAO was defined by comparing the age at the first (hypo)manic and first depressive episode or using the polarity variable provided by the cohort. Specifically, patients were divided into three subgroups:

- (hypo)mania before depression (PAO-M);
- depression before (hypo)mania (PAO-D); and
- mixed (PAO-X).

The third category included patients with mixed episodes and those with a first (hypo)manic and depressive episode within the same year (Table 1). In the primary analysis, we combined patients with (hypo)mania and mixed onset and assigned this as the reference category. In secondary analyses, we excluded the patients in the mixed group.

### Phenotypic disease characteristics

We performed phenotypic analyses of disease onset in patients with bipolar disorder type I from three cohorts: the Dutch Bipolar cohort (*n* = 1313)^22^ and the German PsyCourse^23^ and FOR2107^24^ cohorts, which were analysed jointly (*n* = 346). We analysed the following disease characteristics, which were previously reported as being associated with disease onset and were assessed in a similar way across cohorts: lifetime delusions, lifetime hallucinations, history of suicide attempt, suicidal ideation, current smoking, educational attainment, living together with a partner, and frequency of manic and depressive episodes per year. For more detailed information, see the Supplementary Note 2 and Supplementary Table S9.

### Quality control and imputation of genotype data

The cohorts were genotyped according to local protocols. Individual genotype data of all discovery-stage cohorts were processed with the PGC Rapid Imputation and Computational Pipeline for GWAS (RICOPILI) with the default parameters for standardised quality control, imputation and analysis. Before imputation, filters for the removal of variants included non-autosomal chromosomes, missingness ≥0.02, and a Hardy–Weinberg equilibrium test *P* < 1 × 10^−10^. Individuals were removed if they showed a genotyping rate ≤0.98, absolute deviation in autosomal heterozygosity of *F_het_* ≥0.2, or a deviation >4 s.d.s from the mean in any of the first eight ancestry components within each cohort. From genetic duplicates and relatives (pi-hat >0.2) across all samples, only the individual with more complete phenotypic information on AAO and PAO, gender and diagnosis was retained. Imputation was performed by IMPUTE2 with the Haplotype Reference Consortium reference panel.

### PGS

We calculated PGS based on prior GWAS of attention-deficit hyperactivity disorder (ADHD), autism spectrum disorder (ASD), bipolar disorder, educational attainment (measured as ‘years in education’), major depression (MD), and schizophrenia (see Supplementary Table S3, which includes references). PGS weights were estimated with PRS-CS(see Supplement), with six scores per GWAS (with φ = 1 × 10^−1^, 1 × 10^−2^, 1 × 10^−3^, 1 × 10^−4^, 1 × 10^−5^, and 1 × 10^−6^). We tested the associations of the PGS with the AAO and PAO by linear and logistic regressions, respectively. Gender, bipolar disorder subtype and the first eight ancestry components were included as covariates. The significance threshold was Bonferroni-corrected for 96 tests (α = 0.05/(6 φ thresholds × 8 traits × 2 phenotypes) = 5.2 × 10^−4^).

### GWASs

We performed a discovery GWAS on the 34 cohorts (n = 12 977) and replication analyses in six additional cohorts with *n* = 2237 patients with bipolar disorder. As a first step, we conducted individual GWAS for each cohort with 40 or more patients using the RICOPILI workflow, using the same covariates as in the PGS analyses. Sample sizes are provided in Supplementary Tables S2 and S7. The resulting GWAS did not show an inflation of test statistics for any of the cohorts, indicating limited population stratification (Supplementary Table S2). Next, we performed a fixed-effects meta-analysis using METAL, combining the cohort-specific GWASs. For the meta-analysis summary statistics, we applied the following variant-level post-quality control parameters: imputation INFO score ≥0.9, minor allele frequency (MAF)  ≥0.05, and successfully imputed/genotyped in more than half of the cohorts.

The primary analyses were AAO (normalised, analysed by linear regression) and PAO (analysed by logistic regression). Secondary analyses included GWASs stratified by AAO definition and continent of origin.

We estimated the power to replicate our initial genome-wide significant finding from the discovery GWAS based on the regression coefficients using the *pwr* package in *R*. Assuming the same effect size and MAF (beta 0.075, allele frequency 0.32) and a standardised phenotype, we had 76% power to detect the effect in our sample size of 2237 at an alpha level of 0.1. For comparison, we had 57% power to detect the effect in our discovery sample, using the more stringent genome-wide significance cut-off.

### Heritability analyses

Next, we assessed the overall variance in AAO and PAO explained by genotyped variants (so-called single-nucleotide variant (SNV)-based heritability, *h^2^_SNV_*). For the only individual cohort with more than 1000 samples, we estimated *h^2^_SNV_* with GCTA GREML. In this case, we validated the robustness of the *h^2^_SNV_* estimate with the mean of 1000 × resampling of 95% of the sample. To estimate the overall heritability of the meta-analysis summary statistics we estimated *h^2^_SNV_* by linkage disequilibrium score regression, for each GWAS with sample size >3000. The 95% CIs were constrained to a minimum of 0 and a maximum of 1.

## Results

### Heterogeneity of AAO and PAO across cohorts

Among the four definitions of AAO across the 34 cohorts, impairment/help-seeking was the most common in Europe and diagnostic interview the most common in North America (Table 1, Fig. 1). Across all cohorts, the median AAO was 21 years (range of medians: 16–30 years; Fig. 1). However, substantial differences in the AAO were observed between subgroups: first, the median untransformed AAO was lower in bipolar disorder type I than in type II (type I, 21 years; type II, 22 years; Kruskal-Wallis test *P* = 1.8 × 10^−4^; Supplementary Table S6).

Second, the AAO was lower when determined by diagnostic interview compared with other phenotype definitions (diagnostic interview, 19 years; impairment/help-seeking, 23 years; pharmacotherapy, 30 years; mixed, 22 years; *P* = 2.96 × 10^−191^). Third, the age was lower in North America compared with Europe (Europe, 24 years; North America, 18 years; and Australia, 19.5 years; *P* = 2.0 × 10^−263^). These differences across continents remained significant when including onset definitions and bipolar disorder subtype in a multivariable regression model, indicating that they are likely partially independent from the assessment strategy (Supplementary Table S6).

The majority of patients reported a depression-first PAO. Patients with depression-first were less frequent in the impairment/help-seeking than in the diagnostic interview category (55% and 60%, respectively; *P* = 4.5 × 10^−4^, Supplementary Fig. S1), but their proportions were similar between Europe and North America (57% and 59%, respectively; *P* = 0.17 test of proportion).

### Analyses of disease characteristics

In a meta-analysis of the Dutch and German samples, earlier AAO was significantly associated with a higher probability of lifetime delusions, hallucinations, suicide attempts, suicidal ideation, lower educational attainment and not living together (Table 2, Supplementary Tables S4 and S5). A later AAO was positively significantly correlated with a higher number of manic and depressive episodes per year (see Tables 3, and the Supplementary Note 2). Moreover, a (hypo)manic onset was significantly associated with a greater likelihood of delusions and more manic episodes per year, whereas a depressive onset was associated with a higher probability of suicidal ideation and lifetime suicide attempts.

**Table 2:** The association of age and polarity at onset with disease characteristics in two European bipolar disorder cohorts

Disease characteristic	AAO					PAO				
	*n*	Odds ratio	95% CI	Unadjusted *P*	Adjusted *P*^a^	*n*	Odds ratio	95% CI	Unadjusted *P*	Adjusted *P*
Delusions	1612	0.71	0.64–0.79	1.61 × 10^−9^	1.45 × 10^−8*^	1298	0.62	0.49–0.79	1.04 × 10^−4^	6.24 × 10^−4*^
Hallucinations	1594	0.83	0.74–0.92	3.5 × 10^−4^	1.40 × 10^−3*^	1290	0.93	0.74–1.17	5.22 × 10^−1^	1.00 × 10^0^
Current smoking	1594	0.98	0.89–1.09	7.50 × 10^−1^	7.50 × 10^−1^	1282	1.12	0.89–1.41	3.39 × 10^−1^	1.00 × 10^0^
Suicidal ideation	1518	0.79	0.71–0.88	2.31 × 10^−5^	1.62 × 10^−4*^	1280	1.68	1.32–2.13	2.11 × 10^−5^	1.48 × 10^−4*^
Suicide attempt	1537	0.78	0.69–0.88	2.73 × 10^−5^	1.64 × 10^−4*^	1262	1.58	1.24–2.02	2.67 × 10^−4^	1.34 × 10^−3*^
Educational attainment	1636	1.17	1.06–1.29	2.77 × 10^−3^	8.31 × 10^−3*^	1319	1.06	0.85–1.33	5.93 × 10^−1^	1.00 × 10^0^
Living together	1357	1.28	1.15–1.44	1.01 × 10^−5^	8.08 × 10^−5*^	–	–	–	–	–

AAO, age at onset; PAO, polarity at onset; *n*, total number of participants from the Dutch and German cohorts.

* *P* < 0.05

a. After Bonferroni–Holm correction.

**Table 3:** The association of age and polarity at onset with manic and depressive episodes in two European bipolar disorder cohorts^a^

Episode	AAO					PAO				
	*n*	Estimate^b^	s.e.	Unadjusted *P*	Adjusted *P*^c^	*n*	Estimate	s.e.	Unadjusted *P*	Adjusted *P*
Number of manic episodes per illness year	1436	0.11	0.03	7.08 × 10^−5^	3.54 × 10^−4*^	1156	−0.42	0.06	4.68 × 10^−13^	3.74 × 10^−12*^
Number of depressive episodes per illness year	1231	0.07	0.03	1.93 × 10^−2^	3.86 × 10^−2*^	1051	0.12	0.06	4.63 × 10^−2^	1.85 × 10^−1^

AAO, age at onset; PAO, polarity at onset; *n*, total number of participants from the Dutch and German cohorts.

* *P* < 0.05

a. The number of manic/depressive episodes was divided by (years of illness) + 1. For secondary analyses of the number of episodes not corrected for the years of illness, see the Supplementary Note 2.

b. Unstandardised beta coefficient.

c. After Bonferroni–Holm correction.

### Associations of PGSs with AAO and PAO

**Fig. 2:**
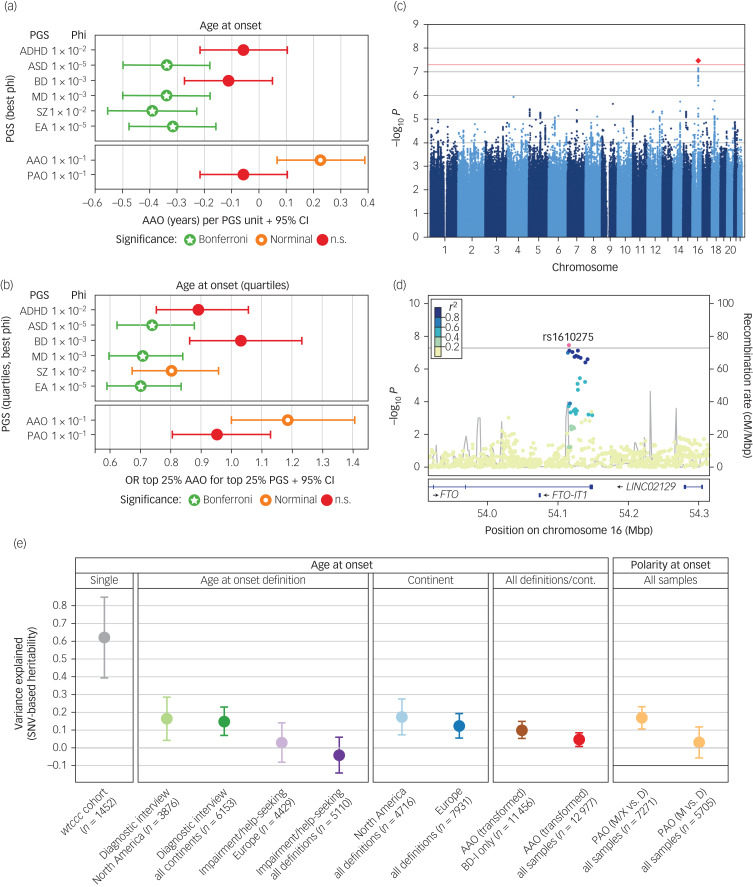
Results from the genome-wide association study (GWAS), polygenic score (PGS) analyses, and heritability analyses. (a) and (b) Results from analyses of PGS. For detailed results, see Supplementary Table S8. Significance levels: n.s., not significant, *P* > 0.05; nominal: *P* < 0.05; Bonferroni, below the Bonferroni-corrected significance threshold corrected for 96 tests (*P* < 5.2 × 10^−4^). (a) Associations of PGSs with the AAO. For interpretability, the plot shows the untransformed AAO. Significance levels are based on the analyses of the AAO after rank-based inverse-normal transformation (which was performed because the distribution of AAO was highly skewed and differed greatly across the study cohorts). (b) Associations of the top versus bottom AAO quartiles with the top versus bottom PGS quartiles. A higher odds ratio (OR) indicates an association with higher AAO. (c) Manhattan plot of the discovery-stage AAO GWAS. (d) Locus-specific Manhattan plot of the top-associated AAO variant. (e) Estimation of the variance in different phenotype definitions explained by genotyped single-nucleotide variants (SNV) (*h^2^_SNV_*). For the cohort *wtccc*, we directly estimated *h^2^_SNV_* from genotype data in GCTA GREML; we estimated all other heritabilities from GWAS summary statistics using LDSC. The plot shows *h^2^_SNV_* estimates and s.e. ADHD, attention-deficit/hyperactivity disorder; ASD, autism spectrum disorder; BD, bipolar disorder; cM, centi Morgan. Mbp, mega base pairs; MD, major depression; EA, educational attainment; SNV, single-nucleotide variant; cont, continent; disorder type I; PAO, polarity at onset; PAO-M, mania/hypomania before depression; PAO-D, depression before mania/hypomania; PAO-X, mixed; SZ, schizophrenia.

Next, we conducted analyses to evaluate whether the genetic liability for five psychiatric disorders and educational attainment were associated with the age at disease onset (Fig. 2(a) and (b) and Supplementary Table S8). After correcting for 96 tests, higher PGSs for ASD (β = −0.34 years per 1 s.d. increase in PGS, s.e. = 0.08, *P* = 9.85 × 10^−6^), major depression (β = −0.34, s.e. = 0.08, *P* = 1.40 × 10^−6^), schizophrenia (β = −0.39, s.e. = 0.08, *P* = 2.91 × 10^−6^) and educational attainment (β = −0.31, s.e. = 0.08, *P* = 5.58 × 10^−5^) were significantly associated with an earlier age at bipolar disorder onset. This was not the case for ADHD or bipolar disorder PGS. No PGS was significantly associated with PAO (Supplementary Fig. S4, Supplementary Table S8).

### GWASs

Next, we attempted to identify individual genetic loci associated with the AAO or PAO. In our discovery GWAS using 34 cohorts, one locus was significantly associated with AAO (rs1610275 on chromosome 16; minor allele G frequency = 0.319, β = 0.075 (s.e. = 0.014), *P* = 3.39 × 10^−8^, Fig. 2(c), Supplementary Table S7, Supplementary Fig. S2). This SNV mapped to an intron of the brain-expressed gene *FTO* (alpha-ketoglutarate dependent dioxygenase, Fig. 2(d)). However, this association was not replicated in an independent sample of six cohorts (Supplementary Table S7, Supplementary Fig. S2). In the replication sample (*n* = 2237), we had 76% power to replicate this SNV at a *P*-value threshold of 0.1. The GWAS of PAO did not yield any genome-wide significant findings, in either primary (PAO-M/-X versus PAO-D) or secondary (PAO-M versus PAO-D) analyses (Supplementary Fig. S3).

We also calculated PGSs for AAO and PAO using leave-one-out summary statistics from these GWASs. The AAO PGS was nominally significantly associated with AAO (β = 0.23 years, s.e. = 0.08, *P* = 0.0087, φ = 0.1, Fig. 2(a) and 2(b)) for five of six tested φ parameters but did not withstand correction for multiple testing (Supplementary Table S8). The PAO PGS was not associated with the PAO (Supplementary Fig. S4).

### SNV-based heritability of the investigated phenotypes

We estimated the SNV-based heritability *h^2^_SNV_* directly from genotype data using GCTA in the only cohort large enough for this analysis, *wtccc*. For the AAO, the *h^2^_SNV_* in *wtccc* was estimated at 0.63 (*P* = 0.0026) (Fig. 2(e)). We evaluated the robustness of this estimate by resampling (mean *h^2^_SNV_* = 0.62, resampling 95% CI 0.15–1.00).

We next estimated *h^2^_SNV_* by linkage disequilibrium score regression (LDSC) from the GWAS summary statistics generated in the present study (Fig. 2(e)). We observed that the heritability decreased when cohorts, phenotype definitions and continents were combined (for example ‘diagnostic interview’ in North America: AAO *h^2^_SNV_* = 0.16, 95% CI 0–0.40, ‘impairment/help-seeking’ in Europe: *h^2^_SNV_* = 0.03, 95% CI 0–0.25, all combined *h^2^_SNV_* = 0.05, 95% CI 0–0.12). As a result of the insufficient sample size, we could not estimate the *h^2^_SNV_* of impairment/help-seeking in North America and diagnostic interview in Europe. For depression versus (hypo)manic and mixed PAO, *h^2^_SNV_* was 0.17 (95% CI 0.05–0.29) on the observed scale.

## Discussion

In our study of bipolar disorder disease onset, we first evaluated the association between AAO or PAO with several clinical indicators of severity in a sample of 1659 patients. We showed that an earlier onset is associated with increased severity, demonstrating and replicating the clinical relevance of these phenotypes. Next, we performed genetic analyses including 12 977 patients from 34 cohorts. Here, we demonstrated that higher genetic risk for ASD, major depression, schizophrenia and educational attainment is associated with an earlier AAO, providing evidence that the age at bipolar disorder onset is influenced by a broad liability for psychiatric illness.

Third, we performed GWAS to identify genetic variants associated with the AAO and PAO, which did not yield any replicated associations. Fourth, we outlined the extent to which age (and, partly, polarity) at onset varies across cohorts, depending both on the continent of recruitment and on the diagnostic instrument used to determine the AAO.

Finally, we showed that this substantial phenotypic heterogeneity affects the heritability of the phenotype, which decreased when multiple cohorts with different diagnostic instruments were combined. This analysis emphasises how genetic analyses are hampered by phenotypic heterogeneity.

### Illness onset is associated with disease course

In a first set of analyses, we confirmed the clinical relevance of disease onset phenotypes in bipolar disorder. Age at bipolar disorder onset was associated with important illness severity indicators, such as suicidality, psychotic symptoms and lower educational attainment, thereby replicating findings of previous studies.^22,25^ Furthermore, patients with a depressive bipolar disorder onset had an increased reported lifetime suicidality, whereas those with a (hypo)manic onset were more likely to experience delusions and more manic episodes per illness year. Contrary to previous evidence in a US (but not in a French) sample, we observed that an earlier onset was associated with fewer episodes per illness year.^26^ Of note, when not normalising for the illness duration, the AAO was, as expected, positively correlated with the number of episodes (see Supplementary Note 2).

### Increased genetic scores for neuropsychiatric phenotypes predict an earlier illness onset

Higher PGSs for schizophrenia, major depression, ASD and educational attainment were significantly associated with a lower AAO, and none of the tested PGSs were significantly associated with PAO. Our findings support the hypothesis that a general liability for psychiatric disorders influences an earlier age of onset in bipolar disorder. Alternatively, an earlier onset may also reflect the broader phenotypic spectrum sometimes captured in early-onset bipolar disorder. Unexpectedly, and in contrast to several other disorders (for example multiple sclerosis), where the strongest genetic risk factors for disease liability are also the most important genetic factors associated with an earlier disease onset,^6,27^ we did not find a significant association between bipolar disorder PGS and the age at bipolar disorder onset. Statistical power may have influenced this result, as the sample sizes of both the schizophrenia and major depression GWASs were larger than that of the bipolar disorder GWAS, improving the predictive ability of these PGSs compared with the bipolar disorder PGS.

The described significant relationship of higher educational attainment PGS with an earlier AAO may seem counterintuitive. However, several studies described a significant association, genetic correlation and causal relationship between a higher educational attainment and bipolar disorder risk.^28,29^ Our findings demonstrate that a high educational attainment PGS is not only a risk factor for bipolar disorder but also associated with an earlier onset of the disorder.

### Lack of replication of the GWAS finding

We have conducted two GWASs to identify individual loci influencing the age and polarity at bipolar disorder onset, possibly independently of affecting lifetime disorder risk. Our discovery GWAS prioritised a genome-wide significant locus associated with the AAO. However, the lack of replication suggests that this finding may have been false-positive. This failure to replicate could have been because of insufficient statistical power in the replication sample, as our power analysis did not account for the likely phenotypic and genetic heterogeneity across cohorts and may thus have underestimated the necessary sample size. Importantly, the replication sample was more ethnically diverse than the discovery sample, which reduced the statistical power. The PAO GWAS, with its lower sample size and dichotomous phenotype, did not identify any genome-wide significant locus.

We also calculated an AAO PGS using our GWAS and tested it on our sample. Although the effect size of this PGS on the AAO was substantial (0.23 years per unit change in the PGS), the association was only nominally significant.

### The heterogeneity of phenotype definitions

A striking finding of our study was the systematic difference in the AAO distribution across cohorts, continents and assessment strategies. Although the assessment strategies varied considerably by continent, with diagnostic interview being mainly used in North America and impairment/help-seeking in Europe, we showed that the continent-level differences were partially independent from the AAO assessment strategy and that both factors contributed significantly to the heterogeneity (Supplementary Table S6). However, variations in the demographic structure of analysed populations may have biased the assessed AAO of bipolar disorder, contributing to the observed differences. Although prior research has identified AAO differences across continents (for example the incidence of early-onset bipolar disorder is higher in the USA than in Europe)^30^ this study is the first to systematically assess this heterogeneity across many cohorts with different ascertainment strategies.

For the polarity at disease onset, the relative proportion of patients reporting a depressive index episode did not differ across continents but across instruments. A (hypo)manic onset was more common if the onset was based on an impairment/help-seeking instead of diagnostic interview phenotype definition.

### Phenotypic heterogeneity affects genetic analyses

Interestingly, the systematic differences in AAO phenotypes across cohorts are reflected in heritability estimates: we observed the highest SNV-based heritability *h^2^_SNV_* when onset was established by diagnostic interview and the lowest when it was captured with more health system-specific and subjective measurements, such as item 4 of the Operational Criteria Checklist for Psychotic Illness (impairment/help-seeking). Moreover, *h^2^_SNV_* estimates approached zero when all samples were combined in our primary analysis (*h^2^_SNV_* = 0.05; 95% CI 0–0.12), underscoring the strong impact of phenotypic heterogeneity. For PAO-M/-X versus PAO-D, we observed significant *h^2^_SNV_* estimates, demonstrating that genetic factors contribute to the polarity at bipolar disorder onset.

Thus, we not only showed systematic heterogeneity in a clinically relevant psychiatric phenotype across cohorts but also provided direct evidence for how this heterogeneity can hamper genetic studies. Similarly, a recent investigation demonstrated that the phenotyping method (for example diagnostic interview versus self-report) significantly influenced heritability estimates, GWAS results and PGS performance in analyses of major depression susceptibility, with broader phenotype definitions resulting in lower heritability estimates.^31^ These results indicate that although increasing samples sizes generally improves the power to detect significant associations, larger samples are no silver bullet: careful phenotype harmonisation and uniform recruitment strategies are likely at least as important.

### Limitations

In addition to diverse phenotype definitions originating from different ascertainment methods, as described above, several factors may have limited the cross-cohort comparability of the AAO and PAO. These factors include differences in the definition and ascertainment of the age at bipolar disorder onset and in how bipolar disorder was diagnosed across cohorts and continents. Such differences can lead to bias, affecting genetic analyses. For example, as patients diagnosed with bipolar disorder type II show, on average, later ages at onset than patients with bipolar disorder type I,^32^ differing proportions of bipolar disorder subtypes across cohorts may have an impact on AAO analyses. Therefore, we included the bipolar disorder subtype as a covariate in our genetic analyses to control for this confounder. Still, this cross-cohort heterogeneity has likely reduced our statistical power.

Given that, for all included cohorts, the disease onset phenotypes were assessed retrospectively, measurement errors associated with interrater reliabilities and recall bias may have occurred across cohorts. For example, hypomania was likely underreported, potentially biasing the PAO towards depression. Notably, such potential issues are not specific to the present study but may affect all retrospective analyses of psychiatric phenotypes. Nevertheless, differences in the diagnosis of bipolar disorder and the ascertained phenotypes between cohorts might have exacerbated these problems. Therefore, future studies should focus on compiling clinically more homogeneous, phenotypically better-harmonised data-sets instead of only assembling the largest possible sample.

Furthermore, the rank-based inverse normal transformation of the AAO phenotype may have affected the GWAS and heritability analyses. We conducted this transformation because, first, the original AAO distribution was highly skewed and thus not suitable for linear regression and, second, the AAO differed significantly between cohorts, which could have biased the meta-analysis. However, by transforming the data, only the rank and not the absolute differences in onset between patients was maintained, reducing the interpretability of the phenotype and the genetic effects.

We performed both SNV-level and polygenic score associations using a structured meta-analysis, which mitigates some of the noise introduced by phenotypic heterogeneity. However, we were unable to account for differences in the underlying genetic aetiology of the phenotypes across cohorts. As described above, phenotypic heterogeneity is an important limitation of our study and should be considered in future phenotype and genetic analyses. Our results need to be interpreted in light of these limitations.

### Implications

Phenotypes of bipolar disorder onset are clinically important trait measures contributing to the well-known clinical and biological heterogeneity of this severe psychiatric disorder. Genetic analysis of AAO and PAO may lead to a better understanding of the biological risk factors underlying mental illness and support clinical assessment and prediction. Our study provides evidence of a genetic contribution to age and polarity at bipolar disorder onset but also demonstrates the need for systematic harmonisation of clinical data on bipolar disorder onset in future studies.

## Data Availability

The data that support the findings of this study are available from the corresponding author, upon reasonable request.

## Supplementary material

For supplementary material accompanying this paper visit http://dx.doi.org/10.1192/bjp.2021.102. click here to view supplementary material
