# Supplementary material for: Characterisation of age and polarity at onset in bipolar disorder
Source: Br J Psychiatry. 2021 Dec;219(6):659–69. doi: 10.1192/bjp.2021.102 (PMC8636611; doi:10.1192/bjp.2021.102)
Supplement: Supplementary file 1 [file S0007125021001021sup001.zip › S0007125021001021sup001.docx]

**Funding details**

Loes M. Olde Loohuis: NIH K99/R00 MH116115.

Eli Stahl: NIH U01MH109536; E.S. is now employed by the Regeneron Genetics Center.

Andrew McQuillin: Medical Research Council, Grant/Award Numbers: G0500791, G0701007, G0801038, G1000708, G9623693N; Stanley Center for Psychiatric Research at the Broad Institute

Douglas Ruderfer: R01MH116269

Maria Grigoroiu-Serbanescu: UEFISCDI, Romania, several grants

Tim B Bigdeli: NIH MH085548, MH085542, MH104564

Fabian Streit: BMBF grant 01EW1810 ERA-Net Neuron ‘Synschiz’, BMBF grant 01ZX1614G e:Med Integrament

Mark J Adams: Wellcome Trust 104036/Z/14/Z, MRC MC_PC_17209

Rolf Adolfsson: Swedish Research Council (2009-33891-68296-196) and the Swedish Federal Government under the LUA/ALF agreement (ALF; RV-161691)

Ole A. Andreassen: Norwegian Research Council, KG Jebsen Stiftelsen, South-Eastern Norway Health Authority

Ceylan Balaban: BMBF ‘BipoLife’ Subproject TPP1

Frank Bellivier: INSERM (Institut National de la Sante et de la Recherche Medicale - C0829), AP-HP (Assistance Publique des Hopitaux de Paris - RBM0436), Fondation FondaMental (RTRS Sante Mentale), Labex Bio-PSY (Investissements dAvenir program managed by the ANR under reference ANR-11-IDEX- 0004-02).

Antonio Benabarre: Thanks the support of the Spanish Ministry of Science and Innovation (PI17/01122)

Wade Berrettini: R01 MH078156

Evelyn J Bromet: NIH MH085548, MH085542, MH104564

Sven Cichon: European Union Horizon 2020 Research and Innovation Programme (grant 785907 (HBP SGA2)), BMBF grant 01ZX1314Ae:Med Integrament, Swiss National Science Foundation (SNSF) grant 156791

William Coryell: R01 MH078154

Nick Craddock: Wellcome Trust (grant #078901)

David Craig: R01 MH078159

Paul E. Croarkin: National Institue of Mental Health (NIMH) R01 MH113700

Udo Dannlowski: This work was funded by the German Research Foundation (DFG, grant FOR2107 DA1151/5-1 and DA1151/5-2 to UD; SFB-TRR58, Projects C09 and Z02 to UD) and the Interdisciplinary Center for Clinical Research (IZKF) of the medical faculty of Munster (grant Dan3/012/17 to UD).

Franziska Degenhardt: German Federal Ministry of Education and Research (BMBF) within the e:Med programme (grant COMMITMENT) and the EU COST (European Cooperation in Science and Technology) programme (COST Action EnGagE CA17130).

Bruno Etain: INSERM (Institut National de la Sante et de la Recherche Medicale - C0829), AP-HP (Assistance Publique des Hopitaux de Paris - RBM0436), Fondation FondaMental (RTRS Sante Mentale), Labex Bio-PSY (Investissements dAvenir program managed by the ANR under reference ANR-11-IDEX- 0004-02).

Ayman H Fanous: NIH MH085548, MH085542, MH104564

Janice M. Fullerton: National Health and Medical Research Council (Australia) grants 1037196,1063960, 1066177; and The Janette Mary ONeil Research Fellowship

Julie Garnham: Canadian Institutes of Health Research (grant #166098); Dalhousie Medical Research Foundation, Genome Atlantic, Lindsay family fund

Elliot Gershon: R01 MH078153

Tim Hahn: TH was supported by the German Research Foundation (DFG grants HA7070/2-2, HA7070/3, HA7070/4) and the Interdisciplinary Center for Clinical Research (IZKF) of the medical faculty of Munster (MzH3/020/20).

Stephane Jamain: INSERM (Institut National de la Sante et de la Recherche Medicale - C0829), AP-HP (Assistance Publique des Hopitaux de Paris - RBM0436), Fondation FondaMental (RTRS Sante Mentale), Labex Bio-PSY (Investissements dAvenir program managed by the ANR under reference ANR-11-IDEX- 0004-02).

Esther Jimenez: EJ thanks the support of the Spanish Ministry of Science and Innovation (PI15/00283, PI18/00805) integrated into the Plan Nacional de I + D + I and co-financed by the ISCIII-Subdireccin General de Evaluacin and the Fondo Europeo de Desarrollo Regional (FEDER); the Instituto de Salud Carlos III; the CIBER of Mental Health (CIBERSAM); the Secretaria dUniversitats i Recerca del Departament dEconomia i Coneixement (2017 SGR 1365), the CERCA Programme, and the Departament de Salut de la Generalitat de Catalunya for the PERIS grant SLT006/17/00357.

Ian Jones: Wellcome Trust (grant #078901)

Lisa Jones: Wellcome Trust (grant #078901)

John R. Kelsoe: R01 MH078151

Tilo Kircher: This work was funded by the German Research Foundation (DFG, grant FOR2107 KI588/14-1 and FOR2107 KI588/14-2 to TK)

George Kirov: Recriutment of the Bulgarian Trios was funded by the Janssen Research Foundation

James A Knowles: NIH MH085548, MH085542, MH104564

Thorsten Kranz: BMBF ‘BipoLife’ Subproject TPP1

Trine Vik Lagerberg: Norwegian Research Council (grant #288542)

Mikael Landen: The Stanley Center for Psychiatric Research, Broad Institute from a grant from Stanley Medical Research Institute, the Swedish Research Council (2018-02653), the Swedish foundation for Strategic Research (KF10-0039), the Swedish Brain foundation (FO2020-0261), and the Swedish Federal Government under the LUA/ALF agreement (ALF 20170019, ALFGBG-716801).

William Lawson: R01 MH078161

Marion Leboyer: INSERM (Institut National de la Sante et de la Recherche Medicale - C0829), AP-HP (Assistance Publique des Hopitaux de Paris - RBM0436), Fondation FondaMental (RTRS Sante Mentale), Labex Bio-PSY (Investissements dAvenir program managed by the ANR under reference ANR-11-IDEX- 0004-02).

Dolores MAlaspina: NIH MH085548, MH085542, MH104564

Melvin McInnis: R01 MH078162

Andrew M McIntosh: Wellcome Trust 104036/Z/14/Z, MRC MC_PC_17209

Helena Medeiros: NIH MH085548, MH085542, MH104564

Ingrid Melle: ‘Regional Health Authority South-Eastern Norway (grants #

2015088,2018093)’

Vihra Milanova: Recriutment of the Bulgarian Trios was funded by the Janssen Research Foundation

Philip B. Mitchell: National Health and Medical Research Council (Australia) grants 1037196, 1177991

John Nurnberger: R01 MH078152

Carlos Pato: NIH MH085548, MH085542, MH104564

Michele T Pato: NIH MH085548, MH085542, MH104564

James B. Potash: R01 MH078157

Mark H Rapaport: NIH MH085548, MH085542, MH104564

Andreas Reif: BMBF ‘BipoLife’ Subproject TPP1

Marcella Rietschel: BMBF grant 01EW1810 ERA-Net Neuron ‘Synschiz’, BMBF grant 01EW1904 ERA-Net Neuron ‘Embed’, BMBF grant 01ZX01909A e:Med SysmedSUD, BMBF grant 01ZX1614G e:Med Integrament

Gloria Roberts: National Health and Medical Research Council (Australia) grants 1037196

Guy Rouleau: Canadian Institutes of Health Research (grant #166098)

William A. Scheftner: R01 MH078155

Peter R. Schofield: National Health and Medical Research Council (Australia) grants 1037196,1063960, 1176716

Janet L Sobell: NIH MH085548, MH085542, MH104564

John Strauss: Canadian Institutes of Health Research, MOP-172013

Gustavo Turecki: Canadian Institutes of Health Research (grant #166098)

Eduard Vieta: EV thanks the support of the Spanish Ministry of Science and Innovation (PI15/00283, PI18/00805) integrated into the Plan Nacional de I + D + I and co-financed by the ISCIII-Subdireccin General de Evaluacin and the Fondo Europeo de Desarrollo Regional (FEDER); the Instituto de Salud Carlos III; the CIBER of Mental Health (CIBERSAM); the Secretaria dUniversitats i Recerca del Departament dEconomia i Coneixement (2017 SGR 1365), the CERCA Programme, and the Departament de Salut de la Generalitat de Catalunya for the PERIS grant SLT006/17/00357.

John B. Vincent: Canadian Institutes of Health Research, MOP-172013

Jordan W Smoller: R01MH063445

Francis J. McMahon: Funded in part by the Intramural Research Program of the NIMH (ZIA MH002843)

Martin Alda: Canadian Institutes of Health Research (grant #166098); Dalhousie Medical Research Foundation, Genome Atlantic, Lindsay family fund

Till. F. M. Andlauer: German Federal Ministry of Education and Research (BMBF) through the DIFUTURE consortium of the Medical Informatics Initiative Germany (grant 01ZZ1804A) and the Integrated Network IntegraMent, under the auspices of the e:Med Programme (grant 01ZX1614J), as well as the European Unions Horizon 2020 Research and Innovation Programme (grant MultipleMS, EU RIA 733161)

The Collection of the Dutch cohort was funded through NIMH R01MH090553 awarded to Dr. Ophoff.

The collection of the Colombian samples was funded through NIMH R01 MH113078 awarded to Drs. Lopez-Jaramillo, Bearden and Freimer.

The collection of the *BOMA-Australia* sample (*bip_bmau_eur*) was supported by the Australian National Medical and Health Research Council (NHMRC) Program Grant 1037196 and Project Grants 1063960 and 1066177. DNA was extracted by Genetic Repositories Australia, an Enabling Facility that was supported by NHMRC Enabling Grant 401184. We gratefully acknowledge the Janette Mary ONeil Research Fellowship (to JMF). We acknowledge support from NHMRC Investigator Grants (Leadership 3) to PBM (1177991) and PRS (1176716).

Funding for the project was provided by the Wellcome Trust under award 076113, 085475 and 090355.

The work by the French group was supported by INSERM (Institut National de la Sante et de la Recherche Medicale - C0829), AP-HP (Assistance Publique des Hopitaux de Paris - RBM0436), the Fondation FondaMental (RTRS Sante Mentale), and the labex Bio-PSY (Investissements dAvenir program managed by the ANR under reference ANR-11-IDEX- 0004-02).
